# Altered Hierarchical Visual Rhythm Tracking in Early Deafness

**DOI:** 10.34133/research.1427

**Published:** 2026-09-16

**Authors:** Li Shen, Tingwei Yu, Yadi Lan, Ruichen Hu, Jie Chen, Ying Wang, Yi Jiang

**Affiliations:** ^1^ State Key Laboratory of Cognitive Science and Mental Health, Institute of Psychology, Chinese Academy of Sciences, Beijing 100101, China.; ^2^ Department of Psychology, University of Chinese Academy of Sciences, Beijing 100049, China.; ^3^ School of Psychology, Central China Normal University, Wuhan 430079, China.; ^4^ Key Laboratory of Adolescent Cyberpsychology and Behavior (CCNU), Ministry of Education, Wuhan 430079, China.; ^5^ School of Educational Science, Cognition and Human Behavior Key Laboratory of Hunan Province, Hunan Normal University, Changsha 410081, China.

## Abstract

Rhythm perception enables humans to navigate dynamic environments. The auditory system is particularly adept at processing rhythmic temporal structure, and auditory training exerts a greater influence than visual training on visual rhythm perception, leading to the hypothesis that auditory coding may support visual rhythm representation. This raises a fundamental question: whether and to what extent the deprivation of auditory coding capabilities influences visual rhythm processing. To investigate this issue, we assessed cortical tracking of hierarchical visual rhythms in hearing and early deaf participants using electroencephalography (EEG). Participants viewed 2 types of rhythmic sequences: geometric shapes and point-light walkers. Each stimulus type consists of a lower-order basic rhythm defined by stimulus recurrence and a higher-order chunk rhythm built on perceptual or cognitive organization. Results showed that hearing participants exhibited robust cortical tracking of both basic and chunk rhythms across stimuli. In contrast, deaf participants exhibited preserved tracking of basic rhythms but reduced tracking of chunk rhythms. Moreover, compared with the hearing group, the deaf group showed reduced visual–auditory and visual–frontal functional connectivity, and individual differences in connectivity strength were positively correlated with chunk-rhythm tracking. These findings support a hierarchical auditory scaffolding framework, in which auditory experience is selectively associated with higher-order visual rhythm perception through temporal chunking mechanisms.

## Introduction

Rhythms permeate the dynamic world, from the ebb and flow of tides to the alternation of day and night and the recurring patterns of human behaviors such as breathing, walking, and speaking [1]. Crucially, the perception of these rhythmic signals is not a unitary operation but relies on temporal computations across multiple, hierarchically organized timescales. It involves the perception of basic rhythms, which arise from the repetition of individual sensory events, and chunk rhythms, which emerge when consecutive events are grouped into larger temporal units periodically [2,3]. Perceiving hierarchical rhythms enables us to adapt to the temporal structure of the environment, which forms a cornerstone of various cognitive functions, including attention allocation [4], language comprehension [5–7], biological motion perception [8,9], and the emergence of visual awareness [10].

Although rhythmic information is available across sensory modalities, the auditory system plays a privileged role in rhythm perception. Humans can effortlessly synchronize with auditory rhythms, whereas their sensorimotor synchronization with rhythmic visual stimuli is less stable [11]. Moreover, auditory training rather than visual training improves visual rhythm perception [12], and the discrimination of visual temporal sequences is more strongly influenced by auditory than by visual information [13]. These findings are compatible with several theoretical frameworks underlying temporal and sequential processing. For example, supramodal timing mechanisms propose that timing information from different sensory modalities may converge onto shared, modality-independent neural mechanisms [14,15]. Domain-general sequence learning theories emphasize domain-independent mechanisms for extracting sequential structure [16–18]. While these mechanisms may support rhythm processing across modalities, they do not explain why the auditory system exerts a privileged and asymmetric influence on rhythm perception relative to the visual system. To address this gap, another stream of work has proposed that the human brain may represent rhythms using an auditory-like code even when the input is visual, because the auditory system provides a particularly efficient representational format for organizing temporal and sequential information [13,19,20]. This raises a fundamental question: How do individuals lacking experience with auditory codes, such as those who are early deaf, process visual rhythms? The auditory scaffolding hypothesis proposes that early auditory experience may provide a “scaffold” for the development of general cognitive abilities related to the representation of temporal or sequential patterns [21]. Consequently, early auditory deprivation may exert influences beyond auditory perception, potentially affecting non-auditory temporal processing abilities that underlie visual rhythm perception.

Early deafness provides a critical case for examining whether and to what extent auditory experience contributes to visual rhythm processing. While there is little direct evidence on this issue, previous studies have examined temporal sequence processing in deaf individuals. The findings remain mixed, with some reporting impairments [22,23] and others showing preserved performance [24,25]. These discrepancies may partly result from differences in task demands, the availability of verbal rehearsal strategy [25], or sign language acquisition [24]. Because behavioral performance reflects not only temporal processing but also other cognitive and linguistic factors, explicit behavioral measures alone may not be sufficiently sensitive or specific for isolating visual rhythm processing. These limitations highlight the need to investigate the neural coding of visual rhythms without explicit rhythm judgments.

Using electroencephalography (EEG), intracranial EEG (iEEG), and magnetoencephalography (MEG), previous research has demonstrated that neural oscillations align with external rhythms, as revealed by enhanced power or phase-locking at corresponding frequencies [26,27], which is known as the cortical tracking effect. Electrophysiological studies have demonstrated the cortical tracking of hierarchical rhythms, at both chunk and basic levels, across various auditory and visual stimuli [8,9,28–30]. Importantly, compared to basic rhythms, the neural representation of chunk rhythms is more tightly linked to higher-order cognitive functions, such as language comprehension [5,30,31], speech production [32], biological motion perception [8,9], and attention allocation [4]. This body of work highlights the necessity of distinguishing between basic and chunk rhythms when exploring whether and how early auditory deprivation affects visual rhythm processing. To date, however, only one study has compared the cortical tracking of basic rhythms (i.e., visual flickers) between hearing and deaf populations [33], leaving the impact of auditory deprivation on the chunk-rhythm processing largely unexplored.

**Fig. 1.**
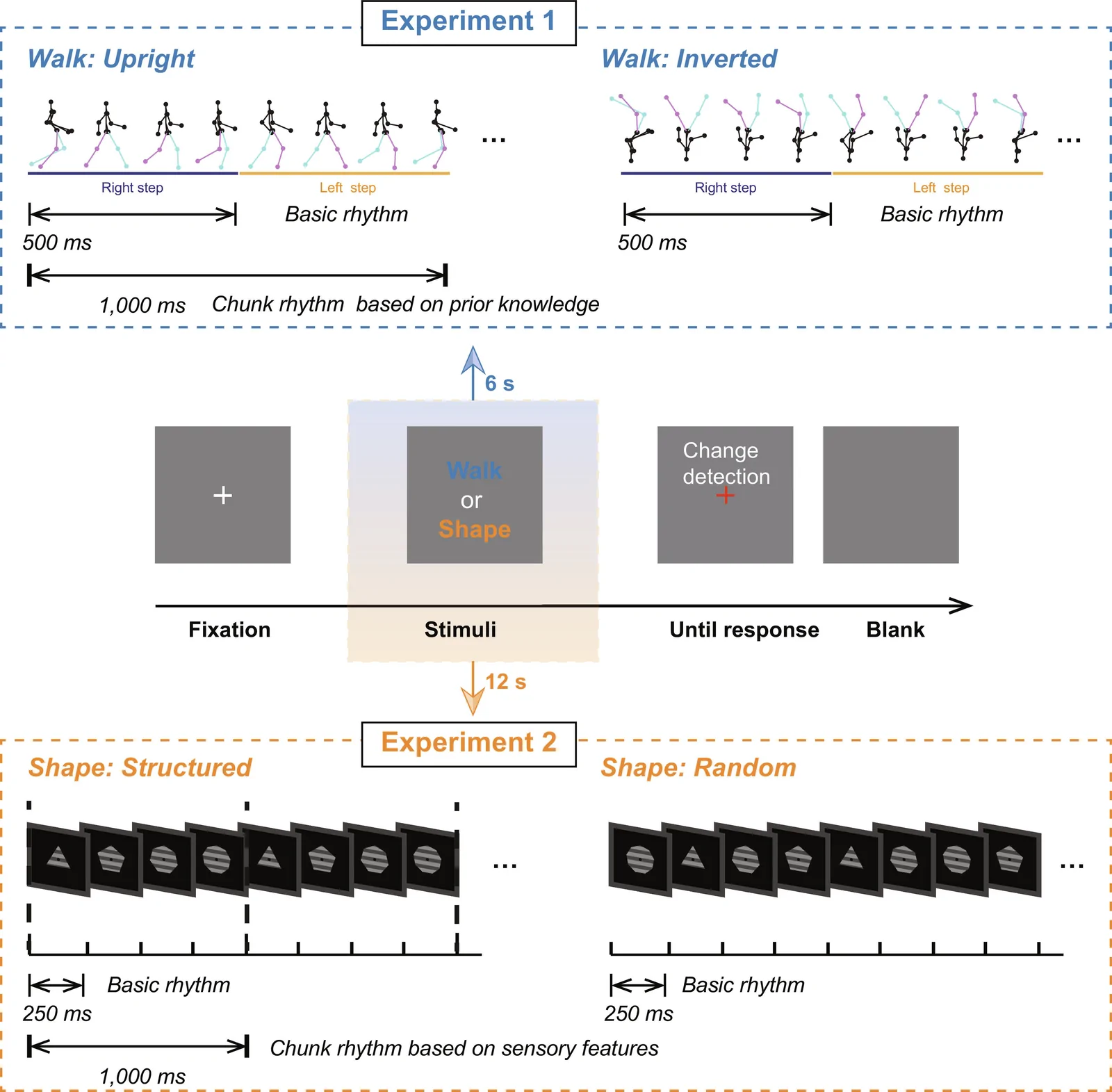
Schematic illustration of stimuli and experimental procedure. The top and bottom panels show representative frames from the walking and geometric shape sequences, respectively. Colors assigned to the dots and connecting lines are for visualization purposes only and were not present in the formal experiment. The middle panel depicts the trial procedure. Each trial began with a fixation, followed by a walk or shape sequence lasting for 6 or 12 s. Brief changes occurred within the stimulus sequence in 17% to 23% of trials, and participants’ task was to detect these changes.

To address these issues, we used EEG to assess cortical tracking of hierarchical visual rhythms (Fig. 1) in hearing and early deaf individuals. Two experiments were conducted using distinct rhythmic stimuli: biological motion sequences in experiment 1 and geometric shape sequences in experiment 2. Both stimuli consisted of a basic rhythm (recurrent footsteps or isochronous presentation of different shapes) and a higher-order chunk rhythm (gait cycles or periodic changes in shapes), although their chunking representations are formed based on prior knowledge and sensory features, respectively. Such manipulations allowed us to explore whether any impairment in deaf individuals generalizes to different higher-order rhythms, besides basic rhythm processing. If early auditory experience scaffolds the development of rhythm processing, we should expect altered cortical tracking of visual rhythms, at either the basic or the chunk level or both, in early deaf participants relative to hearing controls.

To further investigate the neural mechanisms underlying potential group differences, we examined the functional connectivity between the cortical region processing visual inputs (i.e., the visual cortex) and other regions key to rhythm perception while undergoing functional reorganization in deaf individuals. The auditory cortex is particularly relevant in this regard, as both the auditory coding hypothesis and the auditory scaffolding hypothesis emphasize the role of the auditory system in temporal processing. Moreover, previous studies have demonstrated cross-modal plasticity following early deafness, whereby auditory cortical regions are recruited to process information from other sensory modalities, particularly vision [34,35]. In addition, auditory deprivation can delay maturation and alter neural organization in the bilateral prefrontal cortices [36–38]. The inferior frontal cortex (IFC) has been proposed to support sequence chunking [2], a process closely related to the perception of chunk rhythm. Therefore, we investigated whether visual–auditory and visual–IFC connectivity relates to visual rhythm processing in deaf and hearing individuals.

## Results

### Experiment 1: Impaired cortical tracking of chunk rhythm in walking sequences

**Fig. 2.**
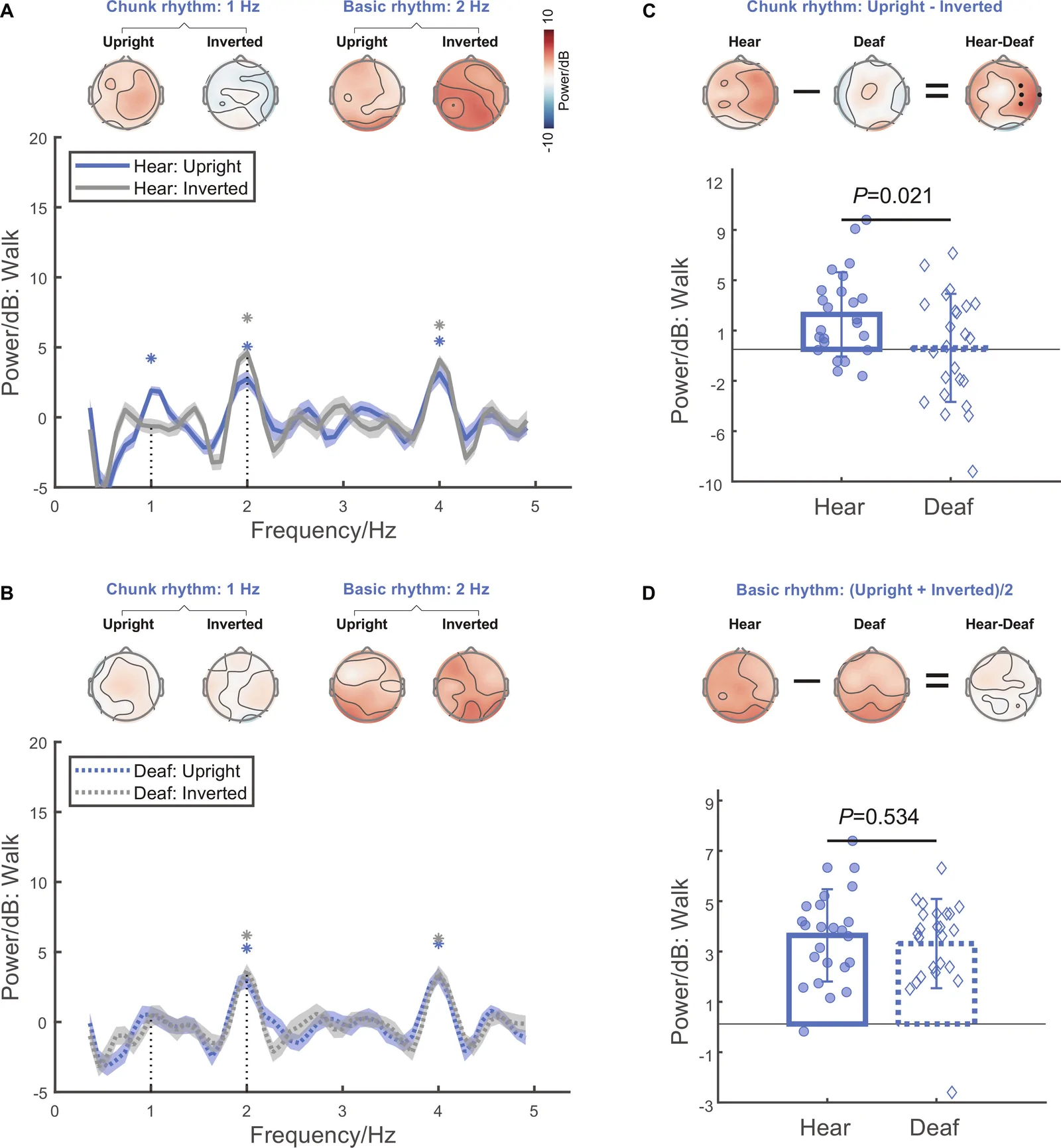
Cortical tracking of basic and chunk rhythms in the walk sequence. Power spectra averaged across all electrodes are shown separately for the hearing group (A) and the deaf group (B). Blue and gray indicate the upright and inverted conditions, respectively. Shaded regions indicate the standard errors (SE) of the mean. Asterisks indicate significant power peaks after FDR correction. Topographic maps above the spectra show the scalp distribution of power at the chunk-rhythm frequency (1 Hz) and basic rhythm frequency (2 Hz) for the upright and inverted conditions. (C) Chunk-rhythm cortical tracking effect, defined as the power difference between the upright and inverted conditions. (D) Basic-rhythm cortical tracking response, defined as the average power across the upright and inverted conditions. In (C) and (D), the topographic maps show the group-averaged scalp distributions of the corresponding power in the hearing group, the deaf group, and the between-group difference. Bar plots show individual data points and group means. Each dot reflects the averaged power across all electrodes from a single participant. Error bars represent one standard deviation (SD) across participants.

When viewing upright walking motion, the hearing group showed significant cortical tracking of both chunk rhythm (1 Hz) and basic rhythm (2 Hz), along with the harmonics (4 Hz), as revealed by significant power peaks at these frequencies [Fig. 2A; 1 Hz: *t* (23) = 6.155, *P* < 0.001, Cohen’s *d* = 1.256; 2 Hz: *t* (23) = 4.657, *P* = 0.001, Cohen’s *d* = 0.951; 4 Hz: *t* (23) = 4.583, *P* = 0.001, Cohen’s *d* = 0.935; false discovery rate (FDR) corrected]. In contrast, significant peaks were observed only at basic rhythm (2 Hz) and its harmonics (4 Hz) under the inverted condition [1 Hz: *t* (23) = −1.158, *P* = 1.000, Cohen’s *d* = −0.236; 2 Hz: *t* (23) = 9.795, *P* < 0.001, Cohen’s *d* = 1.999; 4 Hz: *t* (23) = 11.665, *P* < 0.001, Cohen’s *d* = 2.381; FDR corrected]. The enhanced cortical responses at 1 Hz in the upright rather than the inverted condition, also known as the inversion effect, reflect the spatiotemporal integration of biological motion information. These findings replicated previous evidence that individuals with normal hearing showed hierarchical cortical tracking of rhythms in biological motion while exhibiting an inversion effect only at chunk rhythm [8].

In contrast, deaf participants showed no power peak at 1 Hz in either the upright or inverted condition [Fig. 2B; Upright: 1 Hz; *t* (23) = 0.960, *P* = 0.824, Cohen’s *d* = 0.196; Inverted: 1 Hz; *t* (23) = 0.592, *P* = 1.000, Cohen’s *d* = 0.121; FDR corrected], while they exhibited significant power peaks at 2 Hz and its harmonics (4 Hz) (Fig. 2B; *P*s < 0.001, Cohen’s *d* > 1; FDR corrected). The nonsignificant 1-Hz power peak in the deaf group suggests that cortical tracking of chunk rhythms may be impaired by early auditory deprivation.

Between-group analysis revealed that the inversion effect at 1 Hz (defined as the power difference between the upright and inverted conditions) was significantly reduced in the deaf group relative to the hearing group [Fig. 2C, *t* (46) = 2.396, *P* = 0.021, Cohen’s *d* = 0.692, independent samples *t* test]. A mixed-design analysis of variance (ANOVA), with Group (hearing, deaf) as a between-participants factor and Condition (upright, inverted) as a within-participants factor, also showed a significant Group × Condition interaction at 1 Hz [*F* (1,46) = 5.740, *P* = 0.021, *ƞ_p_*^2^ = 0.111]. According to a cluster-based permutation test, the significant group differences were observed in the right temporo-parietal regions (*P* = 0.012, FC4, C4, T8, CP4). No group difference was observed at 2-Hz response, which was quantified as the average power across the upright and inverted conditions [Fig. 2D, *t* (46) = 0.626, *P* = 0.534, Cohen’s *d* = 0.181, independent samples *t* test]. These results suggest that the lack of auditory experience is associated with the cortical tracking of chunk-level rhythmic structures in biological motion.

At the behavioral level, the deaf group (mean = 88.13% ± 9.04%) also showed significantly lower accuracy in detecting change compared to the hearing group (mean = 97.21% ± 6.12%), *t* (46) = 4.081, *P* < 0.001.

### Experiment 2: Reduced cortical tracking of chunk rhythm in shape sequences

The chunk rhythm in walking motion stimuli is constructed by prior long-term knowledge of locomotion, particularly that walking entails rhythmic alternation between left and right legs [2]. To investigate whether the findings obtained with biological motion could be generalized to chunk rhythm constructed via other rules, we employed rhythmic shape sequences. The chunk rhythm in shape sequences emerges from regular changes in sensory features rather than prior knowledge.

**Fig. 3.**
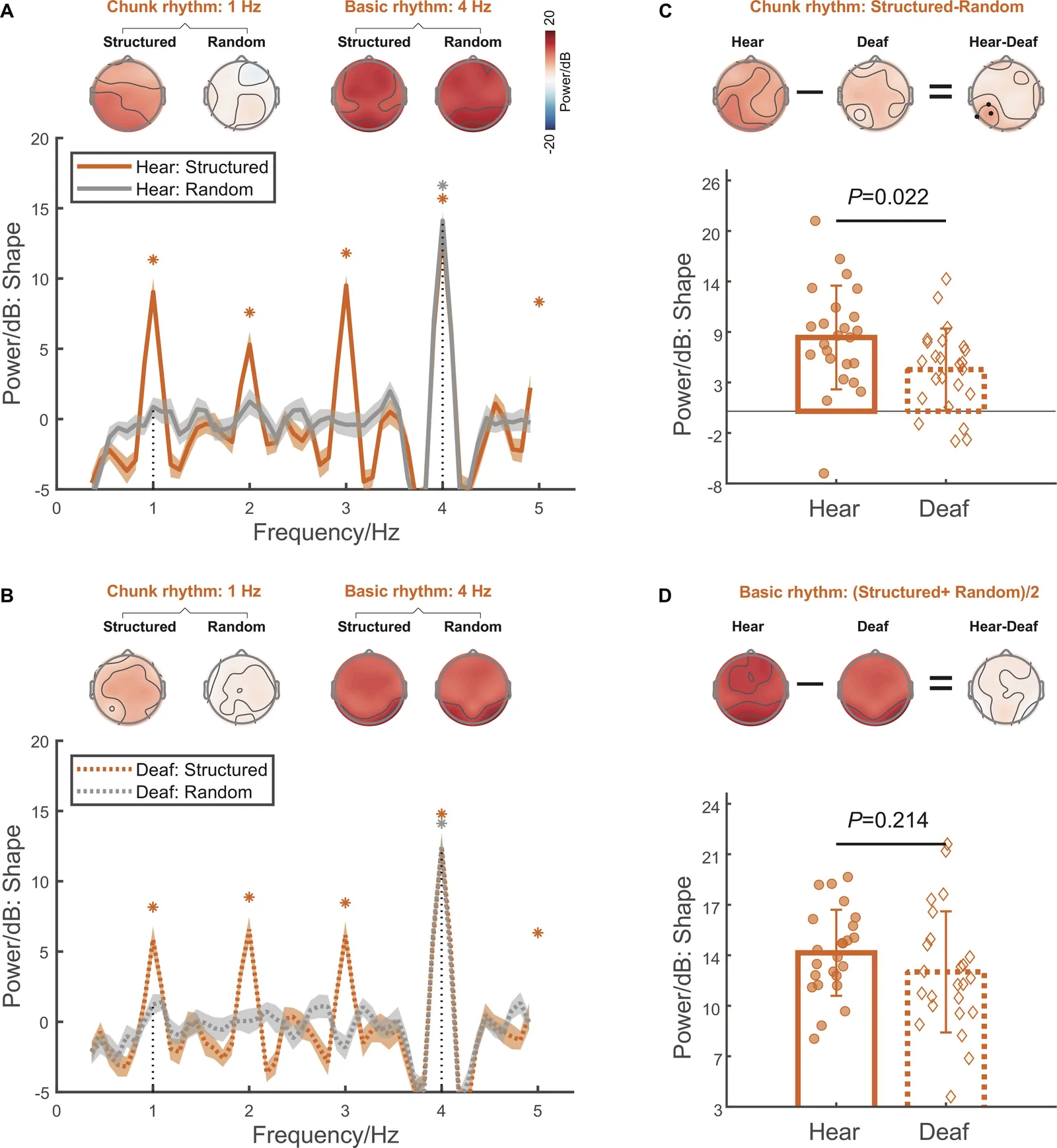
Cortical tracking of basic and chunk rhythms in the shape sequence. Power spectra averaged across all electrodes are shown separately for the hearing group (A) and the deaf group (B). Orange and gray indicate the structured and random conditions, respectively. Shaded regions indicate the standard errors (SE) of the mean. Asterisks indicate significant power peaks after FDR correction. Topographic maps above the spectra show the scalp distribution of power at the chunk-rhythm frequency (1 Hz) and basic rhythm frequency (4 Hz) for the structured and random conditions. (C) Chunk-rhythm cortical tracking effect, defined as the power difference between the structured and random conditions. (D) Basic-rhythm cortical tracking response, defined as the average power across the structured and random conditions. In (C) and (D), the topographic maps show the group-averaged scalp distributions of the corresponding power in the hearing group, the deaf group, and the between-group difference. Bar plots show individual data points and group means. Each dot reflects the averaged power across all electrodes from a single participant. Error bars represent one standard deviation (SD) across participants.

As shown in Fig. 3A, hearing participants showed cortical tracking of both chunk rhythm and basic rhythm in the structured condition [1 Hz: *t* (23) = 9.481, *P* < 0.001, Cohen’s *d* = 1.935; 4 Hz: *t* (23) = 16.858, *P* < 0.001, Cohen’s *d* = 3.441; FDR corrected]. There were also significant power peaks at the harmonics of chunk rhythm (2, 3, and 5 Hz: *P*s < 0.001, Cohen’s *d* > 1; FDR corrected). Under the random condition, only peaks at basic rhythm were observed [4 Hz: *t* (23) = 24.023, *P* < 0.001, Cohen’s *d* = 4.904; 1, 2, 3, and 5 Hz: *P*s > 0.8, Cohen’s *d* < 0.3; FDR corrected]. The unique power peak (1 Hz) in the structured but not the random condition reflected the higher-level chunking of individual items based on sensory features.

Similar to the hearing group, the deaf group showed cortical tracking of both basic and chunk rhythms, along with their harmonics, in the structured condition, and only basic rhythm in the random condition (Fig. 3B; 1 to 5 Hz at structured condition: *P*s < 0.004, Cohen’s *d* > 0.78; 4 Hz at random condition: *P* < 0.001, Cohen’s *d* = 2.663; 1 to 3 Hz and 5 Hz at random condition: *P*s > 0.4, Cohen’s *d* < 0.3; FDR corrected).

However, the structure effect at 1 Hz (stronger cortical tracking in the structure condition versus random condition) was significantly reduced in the deaf group compared to hearing controls [Fig. 3C, *t* (46) = 2.374, *P* = 0.022, Cohen’s *d* = 0.685, independent samples *t* test]. A mixed-design ANOVA, with Group (hearing, deaf) as a between-participants factor and Condition (structured, random) as a within-participants factor, also showed a significant Group × Condition interaction at 1 Hz [*F* (1,46) = 5.636, *P* = 0.022, *ƞ_p_*^2^ = 0.109]. According to a cluster-based permutation test, significant group differences were observed in the central-parietal region (*P* = 0.029, CP3, P3, P7). No group difference reached significance at 4-Hz response, which was quantified as the average power across the structured and random conditions [Fig. 3D, *t* (46) = 1.261, *P* = 0.214, Cohen’s *d* = 0.364, independent samples *t* test]. These findings indicate that the impact of early auditory deprivation extends beyond cortical tracking of prior-knowledge-based chunk rhythm, affecting the neural representation of perceptual chunking.

The behavioral accuracy in detecting change was not different between groups [*t* (46) = 0.263, *P* = 0.794], and both groups performed above chance level [Hearing: 67.11% ± 7.59%; *t* (23) = 10.05, *P* < 0.001; Deaf: 66.52% ± 8.02%; *t* (23) = 11.09, *P* < 0.001].

### Comparable impairment at chunk rhythm across stimuli

Given that impaired cortical tracking of chunk rhythms in the deaf group was observed with both walk and shape stimuli, we further examined whether the impairments were comparable between the 2 stimuli. A 2-way ANOVA was performed on the power at chunk rhythm, with Stimulus type (walk versus shape) as a within-participants factor and Group (hearing versus deaf) as a between-participants factor. Results showed significant main effects of stimulus type [*F* (1,46) = 36.120, *P* < 0.001, *ƞ_p_*^2^ = 0.440] and group [*F* (1,46) = 9.66, *P* =0.003, *ƞ_p_*^2^ = 0.174], but no significant interaction [*F* (1,46) = 0.427, *P* = 0.517, *ƞ_p_^2^* = 0.009]. This suggests that the deaf group showed broadly similar reductions in chunk-rhythm tracking, regardless of whether chunking relies on prior knowledge (walk) or sensory features (shape). We further conducted 2 one-sided tests (TOST) equivalence analyses [39] on the Group × Stimulus interaction contrast. The result supported equivalence under a large-effect bound (Cohen’s *d* = ±0.8) but not under small-to-medium equivalence bounds (Cohen’s *d* = ±0.3 or ±0.5). Thus, the present data allow us to reject the presence of a large stimulus-specific modulation of the group effect.

### Distinct influence of auditory experience across basic and chunk rhythms

Additionally, we examined whether the effect of auditory experience differed between basic and chunk rhythms. Given that the walker and shape stimuli showed similar patterns of results, we collapsed across stimulus types and conducted a 2 × 2 mixed ANOVA with Frequency (basic versus chunk) and Group (hearing versus deaf) as within- and between-participants factors. The analysis revealed a significant Frequency × Group interaction [*F* (1,94) = 4.514, *P* = 0.036, *ƞ_p_*^2^ = 0.046]. The post hoc analysis with Bonferroni correction showed that the group difference mainly resulted from chunk rhythm (*t* = 2.888, *P* = 0.029) rather than basic rhythm (*t* = 0.719, *P* = 1.000), indicating that the effect of auditory experience differed between basic and chunk rhythms.

### Chunk-rhythm tracking is associated with visual–auditory connectivity

The above analyses revealed a selective reduction in chunk-rhythm tracking in the deaf group. To investigate whether chunk-rhythm tracking was associated with the interaction between visual input regions and other regions key to rhythm perception while undergoing functional reorganization in deaf individuals, we conducted an exploratory source-level functional connectivity analysis based on predefined regions of interest (ROIs).

Specifically, we assessed the functional connectivity between the visual cortex (V), which processes visual input, and 2 regions involved in rhythm perception: the auditory cortex (A) and IFC [2,19,40–42]. Furthermore, we investigated the potential correlation between functional connectivity and the strength of chunk-rhythm tracking (see more details in Materials and Methods). Given that experiment 1 (walking stimuli) and experiment 2 (shape stimuli) showed similar reductions in chunk-rhythm tracking in the deaf group, the connectivity and correlation analyses were performed on collapsed data from the 2 experiments.

**Fig. 4.**
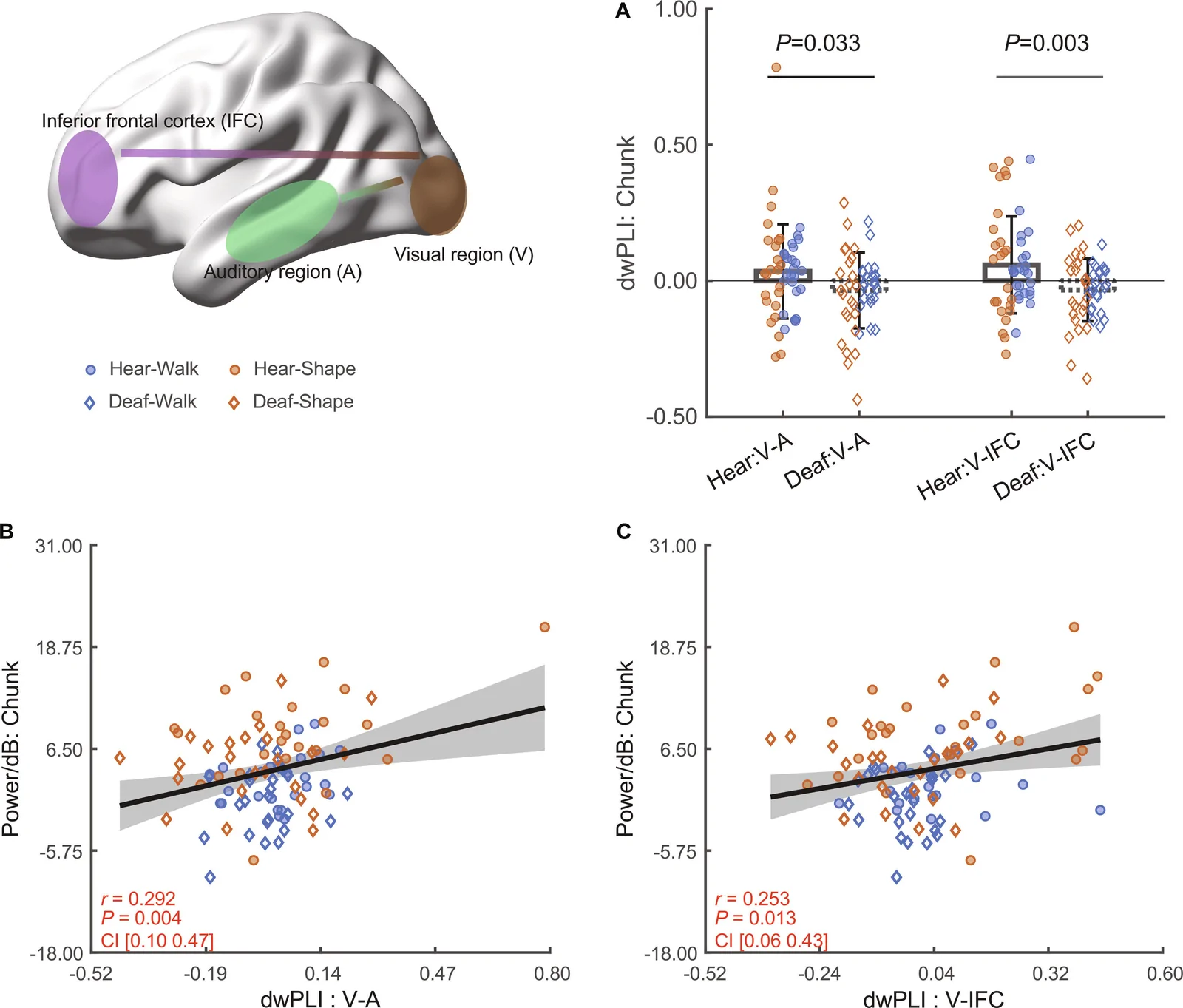
Functional connectivity associated with chunk-rhythm tracking. (A) Chunk-related debiased weighted phase lag index (dwPLI) for visual–auditory (V–A) connectivity and visual–inferior frontal cortex (V–IFC) connectivity. (B and C) Correlations between chunk-related dwPLI and cortical tracking strength. Power/dB on the *y* axis indicates the cortical tracking effect for the chunk rhythm. dwPLI on the *x* axis indicates the functional connectivity. V, visual region (illustrated in brown); A, auditory region (illustrated in green); IFC, inferior frontal cortex (illustrated in purple). Colored dots in the scatterplot represent individual data. Blue symbols indicate the walking stimulus, and orange symbols indicate the shape stimulus. Filled circles represent the hearing group, and open diamonds represent the deaf group. Shaded regions indicate 95% CIs. Error bars indicate one standard deviation (SD) of the mean.

As shown in Fig. 4A, both V–A and V–IFC functional connectivity were stronger in the hearing group than in the deaf group [V–A: *t* (94) = 2.17, *P* = 0.033, Cohen’s *d* = 0.443; V–IFC: *t* (94) = 3.01, *P* = 0.003, Cohen’s *d* = 0.615]. The group difference remained marginally significant [V–A: *t* (93) = 1.91, *P* = 0.060, Cohen’s *d* = 0.391] after removing extreme values. Moreover, both V–A and V–IFC connectivity were positively correlated with the chunk-rhythm tracking effect [Fig. 4B and C; V–A: *r* = 0.292, *P* = 0.004, 95% confidence interval (CI) [0.10, 0.47]; V–IFC: *r* = 0.253, *P* = 0.013, 95% CI [0.06, 0.43]]. These results suggest that connectivity between visual and auditory regions, as well as between visual and IFC regions, is associated with individual differences in the cortical tracking of higher-order visual rhythms. Additional results are presented in Fig. S1.

To examine whether the connectivity–tracking associations could be explained by group or stimulus differences, we fitted separate linear mixed-effects models for V–A and V–IFC connectivity. Each model included functional connectivity, group, stimulus type, and the Group × Stimulus interaction as fixed effects, with a random intercept for participant. The models were estimated using restricted maximum likelihood, with Satterthwaite approximations for the degrees of freedom and 95% percentile bootstrap CIs based on 1,000 samples. After accounting for group, stimulus type, and their interaction, V–A connectivity remained positively associated with chunk-rhythm tracking, *b* = 8.639, *SE* = 2.731, *t* (84.269) = 3.163, *P* = 0.002, 95% bootstrap CI [3.458, 13.850]. V–IFC connectivity was also positively associated with chunk-rhythm tracking, *b* = 6.789, *SE* = 2.946, *t* (86.58) = 2.304, *P* = 0.024, 95% bootstrap CI [1.046, 12.686]. These results indicate that the overall connectivity–tracking associations were not fully explained by group or stimulus differences. Exploratory correlations conducted separately for each group and stimulus type were consistently positive (Fig. S2; see the Supplementary Materials).

## Discussion

As a primary sensory channel for humans, vision provides abundant rhythmic information in everyday life, yet the auditory system is widely regarded as the dominant modality for rhythm perception. Previous research suggests that auditory coding experience may support visual rhythm processing, even when the input is purely visual. To test this hypothesis, the current study used EEG to characterize the influence of early deafness on visual rhythm processing. The results showed that the cortical tracking of chunk rhythms, but not basic rhythms, was reduced in the deaf group compared with the hearing group. These findings are consistent across 2 types of rhythmic stimuli, namely, walk and shape sequences, regardless of whether the chunk rhythm is constructed by long-term prior knowledge or sensory features. Moreover, the V–A and V–IFC functional connectivity were stronger in the hearing group than in the deaf group, and the connectivity strength was associated with individual differences in chunk-rhythm tracking. These findings suggest that deprivation of auditory experience from early life is associated with altered neural processing of visual rhythms.

The spatial distribution of the observed group differences in chunk rhythm differed between the 2 experiments, with a more right-lateralized temporo-parietal distribution for biological motion and a more centroparietal and occipital distribution for shape sequences. This difference may reflect partially distinct perceptual and cognitive processes engaged by the 2 stimuli. Temporo-parietal regions involved in social perception and motion processing are crucial for biological motion perception [43,44], whereas centroparietal and occipital regions have been implicated in the integration of visual feature-based sequences and perceptual organization [45]. Despite these differences in topographical distributions, both stimulus types engage rhythmic structure processing and were similarly affected by early deafness, as reflected in broadly comparable reductions in cortical tracking across experiments and a positive correlation trend between the 2 stimuli within the deaf group (see Fig. S3). Crucially, the observed cluster differences should be interpreted as reflecting the locations of maximal statistical effects rather than exclusive or nonoverlapping neural substrates. Additional analysis revealed that, for walking stimuli, the group differences in chunk rhythm between the 2 clusters identified in the 2 experiments were not significant [*F* (1,46) = 0.971, *P* = 0.330, *ƞ_p_*^2^ = 0.021], indicating a consistent trend of group differences across the 2 clusters.

### Hierarchical auditory scaffolding of visual rhythm perception

The auditory scaffolding hypothesis proposes that early auditory experience serves as a developmental scaffold for temporal and sequential cognition, which would predict that early auditory deprivation leads to a general impairment in visual rhythm processing [21]. While the reduced chunk-rhythm tracking in early deafness compared with the hearing group is broadly consistent with the auditory scaffolding hypothesis, it motivates a possible hierarchical refinement of this framework. The hierarchical auditory scaffolding hypothesis makes a more selective prediction: The association between early auditory experience and visual rhythm processing is not uniform across processing levels but is stronger for higher-order chunk-level organization than for basic-rhythm tracking. Our finding of relatively preserved basic-rhythm tracking and reduced chunk-rhythm tracking in the deaf group provides preliminary evidence for this prediction. However, because the present study did not independently manipulate the temporal integration window and hierarchical complexity, it cannot determine whether the selective chunk-level difference reflects hierarchical organization per se, a longer integration window, or another process associated with chunk formation. Further research is needed to provide a more direct test of the hierarchical auditory scaffolding framework.

The hierarchical auditory scaffolding framework is related to, but distinct from, supramodal timing and cross-modal plasticity accounts. Supramodal timing accounts propose that rhythm processing in the mature brain involves modality-independent neural networks [14,15]. This account is compatible with the observed involvement of the auditory cortex in visual chunk-rhythm tracking in the hearing group. However, it does not predict that deprivation of auditory experience should selectively alter visual rhythm processing, because supramodal timing mechanisms are presumed to operate across sensory modalities regardless of modality-specific experience. Our framework extends this account by suggesting that early auditory experience is associated with the development or calibration of supramodal timing mechanisms, particularly for temporally extended chunk-level organization. Cross-modal plasticity accounts suggest that auditory deprivation can lead to large-scale functional reorganization of cortical systems [34,35]. It provides an important framework for understanding the altered interregional connectivity observed in the deaf group while not specifying how auditory experience contributes to visual rhythm processing in normally hearing individuals. Moreover, cross-modal plasticity is often discussed in terms of compensatory enhancement of visual functions, particularly spatial functions [46], and it does not by itself predict the selective reduction in temporal integration functions, e.g., chunk-rhythm tracking observed here.

This hierarchical account is consistent with prior evidence that rhythm processing is organized across multiple temporal levels in the auditory domain. For example, previous studies have identified distinct neural substrates for lower-level beat and higher-level meter processing [47]: Hearing adults show stronger auditory cortex activation for higher-level meter (i.e., chunk) rhythms than for lower-level beat (i.e., basic) rhythms, while the processing of lower-level beat rhythms preferentially recruits subcortical regions, such as the basal ganglia [48,49]. Notably, the hierarchical organization in the auditory domain is present even in neonates [50]. This early-emerging capability may provide a developmental template for hierarchical rhythm processing in the visual system, establishing a shared organizational principle across sensory modalities.

The preserved basic rhythm perception suggests that basic rhythm (i.e., short timescales) perception is relatively insensitive to early auditory experience. This finding is consistent with the observation that cortical tracking of visual flicker—a typical basic rhythm—was comparable between deaf and hearing signers [33]. Consistent with this view, Zamfira et al. [51] showed that the frequency of rhythmic audio-visual streams—a low-level spectrotemporal property analogous to basic rhythm—systematically narrows temporal binding windows independently of higher-order auditory experience, further corroborating the modality-general and experience-independent nature of basic rhythm processing. In contrast, perceiving chunk rhythm requires temporal integration of multiple sensory events over longer timescales, a process heavily reliant on the auditory system [52–54]. This difference in temporal integration demands raises the further question of whether basic- and chunk-rhythm processing also differ in their dependence on conscious access. Low-frequency cortical oscillations can entrain to subthreshold auditory rhythms [55], while visual studies have revealed awareness-dependent differences in attentional sampling [56], social orienting [57], and associative learning [58]. Future work should test whether subthreshold visual rhythms can be tracked at both levels and whether the selective association between early auditory experience and chunk-level tracking persists without awareness.

### Functional connectivity correlates with chunk-rhythm tracking

The connectivity results provide further insight into the neural mechanisms that may support higher-order visual rhythm tracking and its impairment in early deafness. We found that both V–A and V–IFC functional connectivity correlated with chunk-rhythm tracking effects. These findings suggest that higher-order visual rhythm processing may rely not only on visual encoding per se but also on its functional coupling with a broader temporal–frontal network supporting visual rhythm processing and higher-level structure extraction across sensory modalities [19,48,59]. Recent psychophysical evidence on audiovisual temporal binding also provides indirect support for this interpretation, showing that cross-modal temporal alignment is shaped by rhythmic and spectrotemporal properties of sensory inputs [51]. In the hearing group, the V–A and V–IFC connectivity may support higher-order temporal organization, thereby facilitating the extraction of chunk-level rhythmic structure. In early deaf individuals, however, reduced functional connectivity may reflect a reorganization of the neural network, which could contribute to the reduced cortical tracking of chunk rhythms observed in the deaf group.

Together, these findings support a hierarchical auditory scaffolding framework in which early auditory experience may provide a layered developmental foundation for multi-level temporal integration across modalities. By showing that early deafness is selectively associated with reduced higher-order visual rhythm processing and altered underlying neural architecture, the current study sheds light on how the brain constructs temporal structure and how this process is shaped by early sensory experience.

### Limitations

Several limitations should be noted. First, although the source-level analysis provides an anatomically informed estimate of connectivity, source localization based on 30-channel EEG has limited spatial precision. Given the exploratory nature of the source-level connectivity analysis, these findings should be interpreted as preliminary rather than confirmatory. Moreover, the connectivity measures used here were nondirectional and therefore cannot determine whether auditory regions influence visual rhythm processing, whether visual regions drive auditory responses, or whether the observed coupling reflects bidirectional interactions. The connectivity–tracking associations also warrant caution. Although the linear mixed-effects models indicated that the functional connectivity was associated with chunk-rhythm tracking after accounting for group and stimulus type, the subgroup correlations exhibited mostly nonsignificant trends (Fig. S2). The pooled correlations should therefore not be interpreted as robust within-group individual-differences associations and should be replicated in larger samples. Future studies using methods with higher spatial resolution and greater sensitivity to directional interactions, such as MEG, or high-density EEG combined with effective connectivity analyses, and larger sample sizes will be needed to more precisely characterize the spatial organization and directionality of these interactions. Second, although filtering and independent component analysis (ICA) were applied to reduce slow drifts and common physiological artifacts, no trial-level epoch rejection was performed in experiment 2. Residual slow drifts or transient movement-related artifacts may have influenced the low-frequency spectral estimates.

In addition, although the group difference in auditory experience provides a theoretically meaningful basis for our interpretation, the current design makes it hard to dissociate the specific contribution of auditory deprivation from preexisting neural differences or broader compensatory reorganization associated with early deafness. Sign-language experience is a potential contributing factor to the group differences. Sign languages contain rich temporal and hierarchical organization, including prosodic grouping, phrasal structure, and coordinated rhythmic patterns [60]. Signers show stronger frontal cortical tracking of the low-frequency visual dynamics in sign language than nonsigners [61]. Their experience of sign languages is associated with altered visual temporal-frequency tuning, rather than a general enhancement of cortical responses across frequencies [33]. Such experience might help maintain sensitivity to hierarchical visual structure, potentially attenuating the observed difference between deaf and hearing individuals, and may also contribute to experience-dependent neural reorganization. The absence of a significant association between sign-language acquisition age and chunk-rhythm tracking (see Fig. S4) does not exclude a contribution of sign-language experience. The current design cannot distinguish this from the influence of auditory experience. Future studies should compare deaf signers, hearing signers, and hearing nonsigners while carefully characterizing and accounting for overall language access. Besides sign-language experience, the deaf group was also heterogeneous in several other dimensions, including hearing-loss severity and age of deafness onset. Exploratory individual-difference analyses showed no significant associations between these variables and chunk-rhythm tracking within the deaf group (see Fig. S4). We interpreted these null findings cautiously because of the modest sample size and uneven subgroup distributions. Future studies with larger samples and more balanced subgroup distributions are needed to further address these questions.

## Materials and Methods

### Participants

Twenty-four individuals with congenitally or early deafness (14 female, mean age ± SD = 21.42 ± 1.53 years) and 24 hearing individuals (10 female, mean age ± SD = 20.44 ± 1.62 years) took part in this study. The sample size was determined based on previous comparable EEG studies on rhythm perception, cortical tracking, and studies involving deaf or hearing-impaired populations [8,33]. A chi-square test revealed no significant gender difference between the 2 groups (*χ*^2^ = 1.333, *P* = 0.248). The hearing and deaf groups were matched in gender, age, and the degree of education. All participants had normal or corrected-to-normal vision and no history of neurological or psychiatric disorders. They were naïve to the purpose of the study and provided informed consent.

The information about hearing loss and the history of language acquisition in deaf participants was collected by questionnaire (see Table S1). The etiology of hearing loss was categorized as follows: hereditary (*N* = 7), high fever and/or ototoxic drugs (*N* = 9), and other/unknown (*N* = 8). They reported mild (*N* = 2), moderate (*N* = 10), severe (*N* = 8), or profound (*N* = 4) hearing loss occurring before the age of 3 years or even since birth (*N* = 15). None of the deaf participants had ever used hearing aids or cochlear implants. All had no functional spoken language ability, but were signers of Chinese sign language.

### Stimuli and procedure

All participants completed 2 experiments. Experiment 1 used walking motion sequences (Fig. 1, upper panel), and experiment 2 used shape sequences (Fig. 1, lower panel). To control potential confounding effects unrelated to chunk-rhythm tracking, each experiment included a control condition (inverted walkers in experiment 1, random shapes in experiment 2). These control conditions shared identical visual features with the experimental stimuli but lacked the hierarchical temporal structures. The cortical tracking strength observed in the control conditions can serve as a baseline and be subtracted from that in the experimental conditions, providing a cleaner estimate of chunk-rhythm tracking in both deaf and hearing participants. All stimuli were generated using MATLAB with Psychophysics Toolbox [62,63] and displayed on a 21.5-inch liquid crystal display (LCD) monitor (60 Hz refresh rate) at a viewing distance of 50 cm.

#### Experiment 1 (walk)

For the walking sequences, experimental stimuli were point-light walkers (3.23° × 5.77°) that consisted of 13 point-light dots attached to the major joints and the head of an actor [64]. The point-light stimuli were presented at the center of the screen. Each step lasted 500 ms and formed a basic rhythm at 2 Hz. The alternation between the left and right steps formed a walk cycle of 1 s, i.e., chunk rhythm at 1 Hz, which corresponds to the natural walking speed. In the control condition, we employed inverted walking motion sequences. Previous studies have shown that inversion significantly reduced the cortical tracking of chunk rhythms but not that of basic rhythms [8,9], presumably by disrupting the key kinematic features (e.g., gravity-compatible movements) essential to the chunking of biological motion information.

Each trial in experiment 1 (Fig. 1, middle panel) started with a central fixation (0.44° × 0.44°, 0.8 to 1 s), followed by an upright or inverted point-light walker sequence (6 s). In 17% to 23% of trials, the chunk rhythm was altered once or twice within the stimulus sequence, and participants were required to accurately report the number of changes following the offset of the point-light display. For the once condition, the walker changed speed once, becoming either faster (2 Hz) or slower (0.5 Hz), and then remaining at that speed until the end of the trial. This change occurred randomly between 2 and 3.5 s after stimulus onset. For the twice condition, the walker changed speed to 0.5 Hz or 2 Hz and then returned to the original speed (1 Hz) after 2 to 2.5 s; in these trials, the first change occurred 2 to 2.1 s after stimulus onset. Participants were required to detect and report the number of changes (0, 1, or 2) via key presses as accurately as possible when the point-light display was replaced by a red fixation. After the response, the next trial began 2 to 3.5 s later. Before the formal experiment, participants viewed demos for each change condition and completed 6 practice trials to familiarize themselves with the task. For both upright and inverted conditions, there were 40 trials without changes and 8 to 12 catch trials. The order of upright and inverted conditions was random across participants.

#### Experiment 2 (shape)

For the shape sequences, experimental stimuli were structured sequences composed of gratings in polygonal contours, adopted from a study by Hu et al. [10]. To facilitate the perception of the edges, each polygonal shape (4.43° in circumradius) was superposed on a 5.31° × 5.31° black square. For each trial, grating orientations were randomly set to horizontal or vertical. The gratings were displayed isochronously, 250 ms for each without intervals, forming a basic rhythm at 4 Hz. The edge number of the shape increased or decreased monotonically every 4 items (i.e., triangle → diamond → pentagon → hexagon, or in reverse order), yielding a chunk rhythm at 1 Hz, i.e., chunk cycle. The stimulation frequencies were selected to preserve the natural temporal structure of the walking sequence while maintaining a matched chunk frequency (1 Hz) across stimulus types. For the control condition, stimuli consisted of random sequences created by shuffling the sequence order within each chunk cycle of the structured sequences. These random sequences conveyed only basic rhythms but no chunk rhythms.

Experiment 2 was conducted similarly to experiment 1, except for the following differences. First, considering that participants did not have prior knowledge of the regularity in shape sequences, they might need extra time to perceive the chunk rhythm. Therefore, the stimulus sequence in experiment 2 was extended to 12 s in each trial, and the fixation duration was prolonged to 2 s. Accordingly, the trial number was decreased to ensure that the duration of the experiment would not be too long. Each stimulus condition consisted of 18 experimental trials and 6 catch trials. Second, catch trials contained brief deviations from the regularity of the shape sequence. Specifically, for catch trials in the structured condition, one chunk cycle was randomly selected from the 6th to the 12th chunk cycle, and the third or fourth shape in that cycle was replaced by a novel polygon whose number of edges did not appear in the regular sequence. For catch trials in the random condition, one of 6 consecutive 1-s windows between 6 and 12 s after stimulus onset was randomly selected (6 to 7, 7 to 8, 8 to 9, 9 to 10, 10 to 11, or 11 to 12 s), and the 4 randomly generated shapes presented within that window were replaced by a chunk cycle consisting of 4 shapes whose edge numbers increased or decreased monotonically.

### EEG recording and preprocessing

For both experiments, EEG signals were recorded at 1,024 Hz using a Grael 4K amplifier (Neuroscan) with 30 scalp electrodes arranged according to the international 10–20 system. Bipolar channels were used to record vertical and horizontal electrooculogram signals, with electrodes positioned above and below the left eye’s midpoint and the outer canthi of each eye. Impedances were kept below 10 kΩ for all electrodes.

Data from catch trials were excluded prior to EEG analysis. Preprocessing and subsequent analysis were conducted using the EEGLAB toolbox [65] within MATLAB. EEG data were band-pass filtered between 0.1 and 30 Hz, down-sampled to 500 Hz, and then epoched from 1 s before stimulus onset to stimulus offset (7 s in experiment 1 and 13 s in experiment 2). Epochs with excessive noise were removed via visual inspection. For experiment 1, the proportion of rejected epochs was comparable between upright and inverted conditions, regardless of hearing group [Upright = 3.75%; Inverted = 5.63%; *t* (23) = −1.519, *P* = 0.142] or deaf group [Upright = 5.63%; Inverted = 4.17%; *t* (23) = 0.766, *P* = 0.451]. Moreover, there was no significant group difference in the proportion of rejected epochs (Walk: Hear = 4.69%; Deaf = 4.90%; *t* = −0.088, *P* = 0.931). For experiment 2, no segments were excluded before epoching, and we did not apply trial-by-trial rejection procedures. Therefore, all trials successfully recorded for each participant and condition were retained for subsequent analysis. This decision was motivated by the relatively small number of trials per condition and the longer duration of each trial, which are inherent to the experimental design. Then, independent component analysis [66] via the Runica algorithm [67,68] was applied to eliminate eye and cardiac artifacts. The number of removed components did not differ significantly between groups [experiment 1: Hearing group = 3.33 ± 1.71, Deaf group = 3.33 ± 2.41, *t* (46) = 0, *P* = 1; experiment 2: Hearing group = 2.83 ± 1.24; Deaf group = 2.87 ± 1.51; *t* (46) = −0.10, *P* = 0.92]. The cleaned data were re-referenced to the average mastoids (M1 and M2). EEG data before stimulus onset and the first second following stimulus presentation were removed to minimize the influence of evoked responses on spectral decomposition [69].

### Frequency-domain analysis

The preprocessed EEG signals were first averaged across trials and then converted to the frequency domain via the fast Fourier transform (FFT) with a Hanning window. To match the frequency resolution of experiment 2 (1/11 Hz), zero-padding of length 5,500 was applied to the data in experiment 1. Power spectra were computed as the squared amplitude and then converted to decibels (i.e., 10*log_10_). To remove background noise, the mean power of the 2 adjacent frequency bins on either side was subtracted from each frequency bin [69–72]. This procedure was applied separately for each electrode (except for electrooculogram channels), participant, and condition.

Then, we averaged the power in all electrodes and examined the significance of the cortical tracking effect at each frequency of interest (1 and 2 Hz for experiment 1; 1 and 4 Hz for experiment 2). A right-tailed *t* test was used to assess whether the spectral peak at the target frequency differed significantly from zero. All frequency bins below 5.36 Hz were subjected to the test, and multiple comparisons were corrected using FDR at *P* < 0.05 [73]. Finally, a cluster-based permutation test (5,000 iterations, triangulation-based neighboring electrodes with an average size of 6.2) was used to identify the spatial distribution of electrodes exhibiting significant group difference at *P* < 0.05 [74] based on the FieldTrip toolbox [75].

For the chunk rhythm, group differences were assessed based on the power difference between the experimental condition (upright walkers or structured sequences) and the control condition (inverted walkers or random sequences), because this contrast isolated the higher-order structure-related response. This difference has been commonly used as an intuitive index of the inversion effect or structure effect in previous studies of biological-motion processing and structured-sequence processing [8,10,76,77]. For the basic rhythm, group differences were assessed based on the average power across the experimental and control conditions, because both conditions contained the same basic rhythmic structure and showed clear power peaks at the basic-rhythm frequency.

### Neural connectivity and correlation analysis

We conducted an exploratory ROI-based source-level connectivity analysis in FieldTrip [75]. A source-level approach was chosen because sensor-level EEG connectivity can be affected by volume conduction, reference-dependent effects, and spatial mixing across electrodes. In addition, our hypothesis concerned interactions among anatomically defined regions rather than connectivity between scalp electrodes, making source-level ROI connectivity more appropriate and interpretable, although EEG source-level analysis still has inherent limitations in spatial precision.

Source reconstruction was performed using a standard Boundary Element Method (BEM) head model, a 10-mm template source model, and a Linearly Constrained Minimum Variance (LCMV) beamformer. Three theoretically motivated ROIs were defined in Montreal Neurological Institute (MNI) space based on previous rhythm-related neuroimaging work [48]: the visual cortex (V; left/right: [−24, −94, −2]/[16, −92, −6]), the auditory cortex (A; left/right: [−48, −14, 0]/[44, −20, 8]), and the IFC (left/right: [−36, 10, 26]/[16,18,48]). For each ROI, grid points within a 1.5-cm-radius sphere around the corresponding coordinates and inside the brain were included. A common spatial filter was computed from data pooled across the experimental and control conditions and was then applied separately to each condition to extract ROI-level virtual-channel time series. Frequency-domain representations of the virtual channels were estimated using multitaper Fourier analysis with discrete prolate spheroidal sequence (DPSS) tapers, and functional connectivity between ROI pairs was quantified using debiased weighted phase lag index (dwPLI). To assess the connectivity at chunk frequency, dwPLI values were averaged across the chunk frequency at 1 Hz and its 2 neighboring frequency bins on each side: 0.8182, 0.9091, 1.0000, 1.0909, and 1.1818 Hz [78]. Furthermore, we investigated the potential correlation between the functional connectivity and the strength of chunk-rhythm tracking.

Given that experiment 1 (walking stimuli) and experiment 2 (shape stimuli) showed a similar reduction in chunk-rhythm tracking in the deaf group, the connectivity and correlation analyses were performed on collapsed data from the 2 experiments. Chunk-related connectivity and chunk-rhythm tracking effects were defined as the difference between experimental stimuli and control stimuli: upright minus inverted for the walking stimulus, and structured minus random for the shape stimulus.

## Ethical Approval

The study was approved by the institutional review board of the Institute of Psychology, Chinese Academy of Sciences. All participants provided written informed consent before participation.

## Data Availability

The data supporting the findings of this study are available at https://www.scidb.cn/s/eMNfa2. The original experimental code and raw data can be obtained from the corresponding authors upon reasonable request.
