# Supplementary material for: Altered Hierarchical Visual Rhythm Tracking in Early Deafness
Source: Research (Wash D C). 2026 Sep 16;9:1427. doi: 10.34133/research.1427 (PMC13578771; doi:10.34133/research.1427)
Supplement: Supplementary file 1 [file research.1427.f1.zip › Fig S1.pdf]

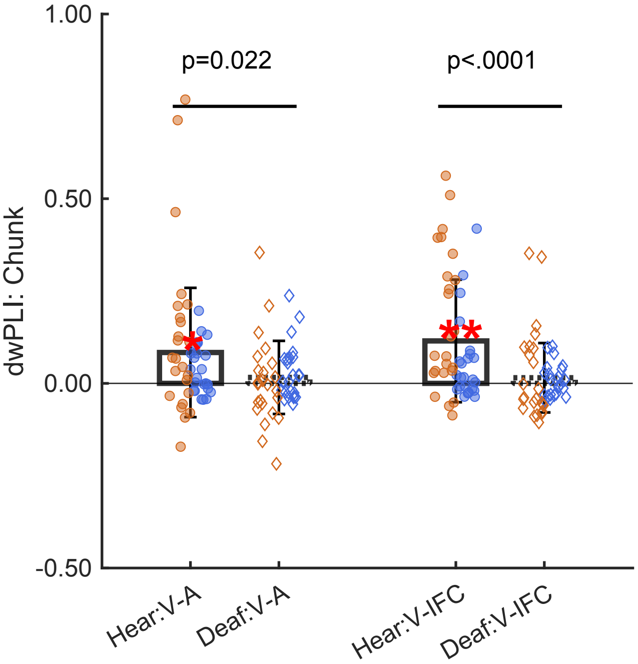

1.00
0.50
0.00
-0.50
dwPLI: Chunk
p=0.022
p<.0001
Hear:V-A
Deaf:V-A
Hear:V-IFC
Deaf:V-IFC
