# Supplementary material for: Altered Hierarchical Visual Rhythm Tracking in Early Deafness
Source: Research (Wash D C). 2026 Sep 16;9:1427. doi: 10.34133/research.1427 (PMC13578771; doi:10.34133/research.1427)
Supplement: Supplementary file 1 [file research.1427.f1.zip › Fig S2.pdf]

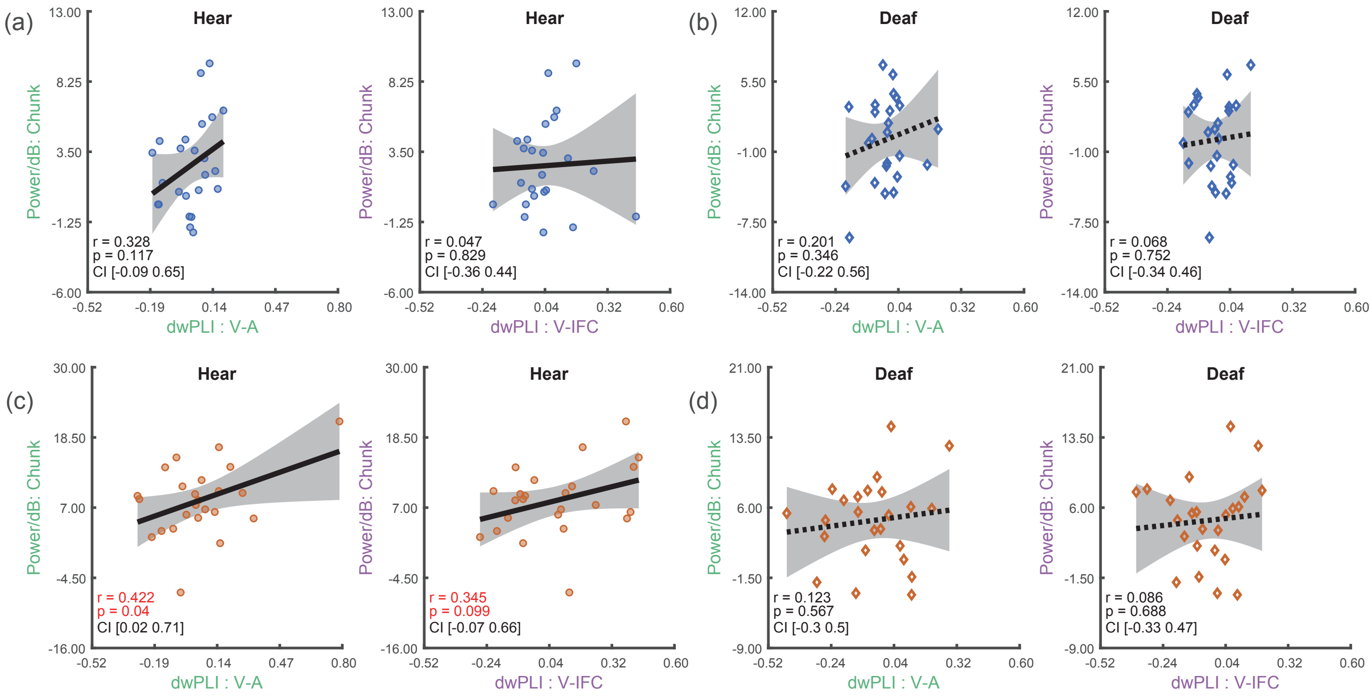
(a)
Hear
r = 0.328
p = 0.117
CI [-0.09 0.65]
Power/dB: Chunk
dwPLI : V-A
Hear
r = 0.047
p = 0.829
CI [-0.36 0.44]
Power/dB: Chunk
dwPLI : V-IFC
(b)
Deaf
r = 0.201
p = 0.346
CI [-0.22 0.56]
Power/dB: Chunk
dwPLI : V-A
Deaf
r = 0.068
p = 0.752
CI [-0.34 0.46]
Power/dB: Chunk
dwPLI : V-IFC
(c)
Hear
r = 0.422
p = 0.04
CI [0.02 0.71]
Power/dB: Chunk
dwPLI : V-A
Hear
r = 0.345
p = 0.099
CI [-0.07 0.66]
Power/dB: Chunk
dwPLI : V-IFC
(d)
Deaf
r = 0.123
p = 0.567
CI [-0.3 0.5]
Power/dB: Chunk
dwPLI : V-A
Deaf
r = 0.086
p = 0.688
CI [-0.33 0.47]
Power/dB: Chunk
dwPLI : V-IFC
