# Supplementary material for: Altered Hierarchical Visual Rhythm Tracking in Early Deafness
Source: Research (Wash D C). 2026 Sep 16;9:1427. doi: 10.34133/research.1427 (PMC13578771; doi:10.34133/research.1427)
Supplement: Supplementary file 1 [file research.1427.f1.zip › Fig S3.pdf]

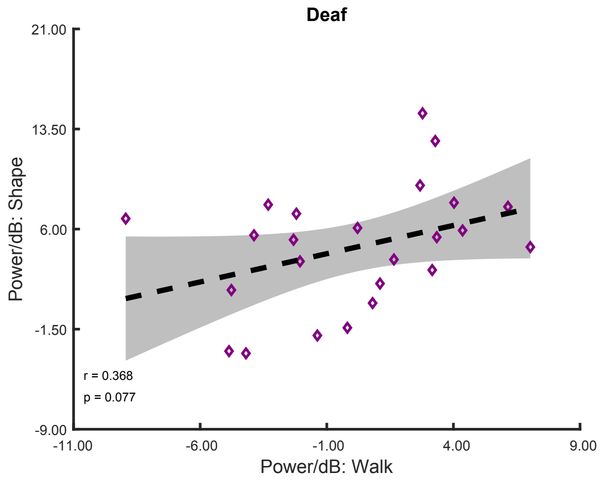

Deaf
21.00
13.50
6.00
-1.50
-9.00
Power/dB: Shape
r = 0.368
p = 0.077
-11.00
-6.00
-1.00
4.00
9.00
Power/dB: Walk
