# Supplementary material for: Altered Hierarchical Visual Rhythm Tracking in Early Deafness
Source: Research (Wash D C). 2026 Sep 16;9:1427. doi: 10.34133/research.1427 (PMC13578771; doi:10.34133/research.1427)
Supplement: Supplementary file 1 [file research.1427.f1.zip › Fig S4.pdf]

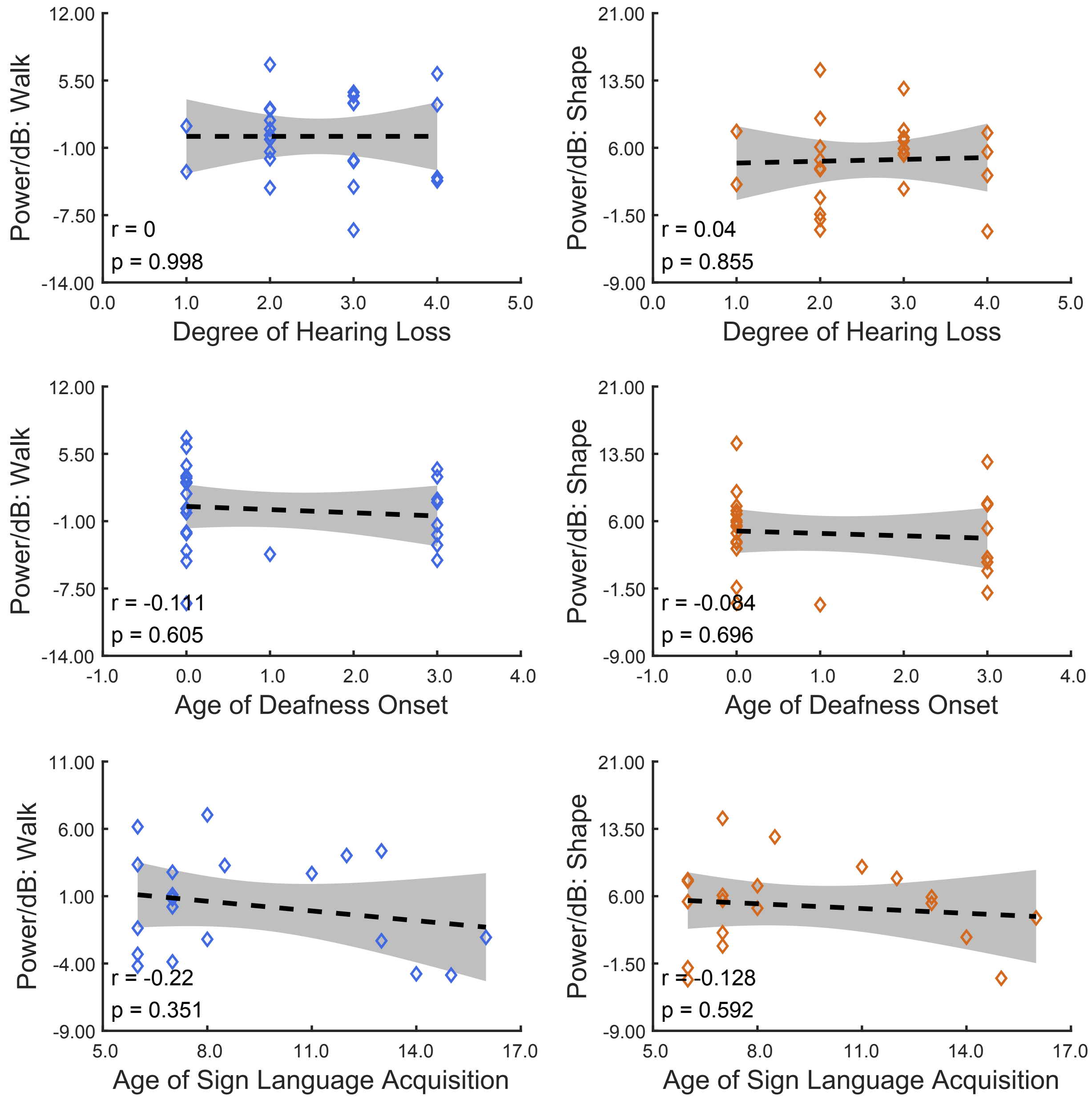

Power/dB: Walk
Degree of Hearing Loss
r = 0
p = 0.998
Power/dB: Shape
Degree of Hearing Loss
r = 0.04
p = 0.855
Power/dB: Walk
Age of Deafness Onset
r = -0.111
p = 0.605
Power/dB: Shape
Age of Deafness Onset
r = -0.084
p = 0.696
Power/dB: Walk
Age of Sign Language Acquisition
r = -0.22
p = 0.351
Power/dB: Shape
Age of Sign Language Acquisition
r = -0.128
p = 0.592
