# Supplementary material for: Altered Hierarchical Visual Rhythm Tracking in Early Deafness
Source: Research (Wash D C). 2026 Sep 16;9:1427. doi: 10.34133/research.1427 (PMC13578771; doi:10.34133/research.1427)
Supplement: Supplementary file 1 [file research.1427.f1.zip › sm.docx]

## Supplementary Materials

### **Characteristics of deaf participants**

| **Table S1. Characteristics of deaf participants** | | | | | | |
| --- | --- | --- | --- | --- | --- | --- |
| Sex | Age  (years) | Age of deafness  (years) | Etiology of hearing loss | Degree of hearing loss | Age of sign language acquisition (years) | Hearing aid use |
| female | 21 | 0 | hereditary | profound | unknown | never used |
| male | 22 | 0 | ototoxic drugs | moderate | 7 | never used |
| male | 19 | 0 | high fever | severe | 6 | never used |
| female | 22 | 0 | hereditary | moderate | 8 | never used |
| male | 23 | 0 | hereditary | severe | 8 | never used |
| male | 20 | 1 | high fever and ototoxic drugs | profound | 6 | never used |
| female | 21 | 3 | other/unknown | severe | 13 | never used |
| female | 22 | 0 | other/unknown | severe | unknown | never used |
| male | 22 | <3 | other/unknown | mild | 7 | never used |
| male | 24 | 0 | hereditary | moderate | 11 | never used |
| female | 21 | 3 | high fever | moderate | 7 | never used |
| male | 21 | 3 | high fever | moderate | 6 | never used |
| female | 23 | 0 | ototoxic drugs | moderate | 15 | never used |
| male | 19 | 0 | high fever | moderate | unknown | never used |
| male | 21 | 3 | high fever | mild | 6 | never used |
| male | 23 | 0 | other/unknown | moderate | 16 | never used |
| male | 18 | 3 | other/unknown | severe | 12 | never used |
| female | 23 | 0 | high fever | moderate | 7 | never used |
| female | 23 | <3 | other/unknown | severe | 14 | never used |
| female | 21 | 3 | high fever | severe | 8.5 | never used |
| male | 20 | 0 | hereditary | moderate | unknown | never used |
| female | 20 | 0 | hereditary | profound | 6 | never used |
| female | 23 | 0 | hereditary | severe | 13 | never used |
| male | 22 | 0 | other/unknown | profound | 7 | never used |

### **The significance of functional connectivity across experimental conditions**

We performed the surrogate data analysis on experimental stimuli (upright walking sequences or structured shape sequences) to examine whether dwPLI values exceed chance levels.

For each participant and condition, a single control surrogate dataset was derived from the original dataset by jittering the onset of each step-cycle or structure-cycle relative to its original onset by a randomly selected integer value drawn from the ranges −500 to −50 ms and 50 to 500 ms, thereby avoiding minimal temporal shifts around the original onset. This procedure removed the consistent relationship between the EEG signal and the stimuli while preserving each epoch’s general timing within the exposure period. Epochs were then extracted based on the surrogate stimulus onsets, and dwPLI was computed using the surrogate data following the same procedure as described in the manuscript.

As shown in **Fig.S1,** the dwPLI in hearing group (V-A: *t* (47) = 2.67, *p* = 0.010, Cohen’s *d* = 0.385; V-IFC: *t* (47) = 3.22, *p* = 0.002, Cohen’s *d* = 0.465) but not deaf group (V-A: *t* (47) = -1.68, *p* = 0.100, Cohen’s *d* = -0.242; V-IFC: *t* (47) = -1.07, *p* = 0.290, Cohen’s *d* = -0.155) significantly exceeded chance levels. Meanwhile, the group differences were significant in V-A connectivity as well as V-IFC connectivity (V-A: *t* (94) = 2.33, *p* = 0.022, Cohen’s *d* = 0.475; V-IFC: *t* (94) = 3.62, *p* < 0.001, Cohen’s *d* = 0.739).

**Fig.S1. The dwPLI in the Upright/Structured condition.** Red asterisk indicates the dwPLI significantly exceeded chance levels (*p* < 0.05). *: p<0.05; **: p<0.01. Error bars indicate one standard deviation (SD) of the mean.

### **The correlation between functional connectivity and chunk-rhythm tracking**

We conducted exploratory correlation analyses separately for each group and stimulus type. For the walking stimulus, the correlations between chunk-rhythm tracking and V–A connectivity were *r* = .328, 95% CI [−.09, .65], *p* = .117, in the hearing group and *r* = .201, 95% CI [−.22, .56], *p* = .346, in the deaf group. The corresponding correlations for V–IFC connectivity were not significant for either group (Hearing: *r* = .047, 95% CI [−.36, .44], *p* = .829; Deaf: *r* = .068, 95% CI [−.34, .46], *p* = .752). For the shape stimulus, V–A connectivity was correlated with chunk-rhythm tracking in the hearing group (*r* = .422, 95% CI [.02, .71], *p* = .040) but not in the deaf group (*r* = .123, 95% CI [−.30, .50], *p* = .567) The corresponding correlations for V–IFC connectivity were marginally significant in hearing group (*r* = .345, 95% CI [−.07, .66], *p* = .099) but not significant in deaf group (*r* = .086, 95% CI [−.33, .47], *p* = .688). It is worth noting that the subgroup sample sizes may provide limited sensitivity to the correlation analysis.

**Fig.S2. Correlation between functional connectivity and chunk-rhythm tracking.** Power/dB on the y-axis indicates the cortical tracking effect for the chunk rhythm. Debiased weighted phase lag index (dwPLI) on the x-axis indicates functional connectivity. V, visual region; A, auditory region; IFC, inferior frontal cortex. Colored dots in the scatter plot represent individual data. Blue symbols indicate the walking stimulus, and orange symbols indicate the shape stimulus. Filled circles represent the hearing group, and open diamonds represent the deaf group. Shaded regions indicate 95% confidence intervals.

### **The correlation between two stimuli in chunk-rhythm tracking effects**

We examined whether the chunk-rhythm tracking effects were correlated across the two types of visual stimuli. Interestingly, within the deaf group, the chunk-rhythm tracking effect for the point-light walker tends to be positively correlated with that for the geometric-shape sequence (*r* = 0.368, *p* = 0.077). Individuals who showed stronger chunk-rhythm tracking for biological motion also tended to show stronger chunk-rhythm tracking for shape-based visual sequences. This cross-stimulus association is consistent with the possibility that the two stimuli may share a common higher-order temporal organization component, the processing of which is impaired in the deaf group.

**Fig. S3. The correlation between shape and walk.** The power is defined as the difference between the upright (structured) and inverted (random) conditions. Shaded regions indicate 95% confidence intervals.

### **The correlation between individual characteristics and chunk-rhythm tracking**

We examined whether individual differences within the deaf group, regarding the severity of hearing loss, age of deafness onset, and age of sign language acquisition, was associated with the magnitude of chunk-rhythm tracking impairment, quantified as the difference between the structured/upright and control conditions. As shown in **Fig. S4**, we did not observe any significant correlation between these variables (*p*s > 0.3). We interpret these null results cautiously because of the modest sample size and uneven subgroup distributions.

**Fig.S4. The correlation between chunk-rhythm tracking and individual participant characteristics**. The power is defined as the difference between the upright (structured) and inverted (random) conditions. Shaded regions indicate 95% confidence intervals. Blue and orange dots indicate walk and shape stimuli, respectively.
